# A Deep Convolutional Neural Network for Prediction of Peptide Collision Cross Sections in Ion Mobility Spectrometry

**DOI:** 10.3390/biom11121904

**Published:** 2021-12-19

**Authors:** Yulia V. Samukhina, Dmitriy D. Matyushin, Oksana I. Grinevich, Aleksey K. Buryak

**Affiliations:** A.N. Frumkin Institute of Physical Chemistry and Electrochemistry, Russian Academy of Sciences, 31 Leninsky Prospect, GSP-1, 119071 Moscow, Russia; juliesam2008@mail.ru (Y.V.S.); oksigrinevich@gmail.com (O.I.G.); akburyak@mail.ru (A.K.B.)

**Keywords:** proteomics, ion mobility spectrometry, deep learning, peptides

## Abstract

Most frequently, the identification of peptides in mass spectrometry-based proteomics is carried out using high-resolution tandem mass spectrometry. In order to increase the accuracy of analysis, additional information on the peptides such as chromatographic retention time and collision cross section in ion mobility spectrometry can be used. An accurate prediction of the collision cross section values allows erroneous candidates to be rejected using a comparison of the observed values and the predictions based on the amino acids sequence. Recently, a massive high-quality data set of peptide collision cross sections was released. This opens up an opportunity to apply the most sophisticated deep learning techniques for this task. Previously, it was shown that a recurrent neural network allows for predicting these values accurately. In this work, we present a deep convolutional neural network that enables us to predict these values more accurately compared with previous studies. We use a neural network with complex architecture that contains both convolutional and fully connected layers and comprehensive methods of converting a peptide to multi-channel 1D spatial data and vector. The source code and pre-trained model are available online.

## 1. Introduction

Rapid proteome profiling is often performed by digesting proteins into peptides, followed by the determination of peptides using tandem mass spectrometry (MS2). This approach is called “bottom-up” proteomics and is extensively used. Highly complex mixtures of relatively short peptides (<100 residues) are analyzed by high-performance liquid chromatography-mass spectrometry (HPLC-MS) in this case. The structure determination based on the high-resolution MS2 spectrum is not always correct and unambiguous [1]. It is a significant challenge to distinguish residues of isoleucine and leucine [2], but even without distinguishing these amino acids, the MS2-based identification of peptides is a difficult task [3].

Ion mobility spectrometry (IMS) is a method of ion separation based on their mobility in the gas phase before mass spectrometric identification [4]. This method adds a new dimension to HPLC-MS analysis. Additional information such as HPLC retention time (RT) and IMS collision cross section (CCS) allows for wrong identifications to be rejected [5] and improves the accuracy of proteomic analysis [6,7]. In order to use the experimental values of RT or CCS, we must have reference or predicted values for the peptide candidate and then compare them with the observed values. Since the reference data for most peptides occurring in the proteomic analysis are absent, the prediction of the RT and CCS of short peptides is an important task. For RT prediction, relatively accurate deep learning-based methods were developed [8,9,10]. The peptide CCS prediction is less studied.

It should also be noted that by using accurate predictions of RT and CCS, it is possible to use one-dimensional MS (MS1) for proteome analysis instead of MS2 [11]. This allows for using much shorter HPLC gradients and determining proteins much faster [12]. The typical HPLC analysis of the digested mixture of peptides (complete proteome analysis) using MS2 takes 30–120 min per sample (duration of chromatographic method). The use of MS1 allows conducting such an analysis in 5–10 min [12].

There are several previous works devoted to the CCS prediction or the IMS drift time prediction [5,13,14,15,16]. Different machine learning approaches were used, e.g., support vector regression [13], linear empirical formulae [14], relatively shallow neural networks with fully connected layers [15], and other methods. Most previous works are based on relatively small data sets, and the data set size limits the application of the deep learning methods. These methods are enormously accurate but require large training sets. Recently, a large (more than 0.5 million data records) data set with information on the CCS values of peptides was released [16]. It opens up an opportunity to develop more versatile and accurate methods for this task using deep learning.

Peptide structure is represented as a sequence of amino acid residues. The two most natural ways to feed 1D sequential data to the deep neural network are: recurrent neural network (RNN) and 1D convolutional neural network (1D CNN). The 1D CNN has a significant drawback: it is mainly sensitive to local features and is much less sensitive to the relative order of local features. The RNN is sensitive to the full structure of a sequence. This unique advantage of the RNN allows it to be used in natural language processing and other tasks. [16] However, the 1D CNN is easier to train and is generally the more robust neural network architecture.

Recently [16], authors of the largest data set of peptide CCS values applied a RNN (two layers with 500 nodes each one) to this task. A median percentage error (MdPE) of 1.4% was achieved. This indicates good accuracy; however, this error is much larger than the error of the experimental data themselves. The median percentage deviation between independently acquired values of 0.6–0.7% was reported in this study. Hence, there is still space for further prediction improvement.

To the best of our knowledge, a 1D CNN has never been applied for the prediction of the CCS of peptides. For another closely related problem, i.e., for the prediction of peptide RT values, solutions based on the 1D CNN have similar or even better accuracy compared with RNN-based solutions [17]. Both architectures can also be used together. A 1D CNN was also applied for the prediction of the CCS of small molecules [18] and the prediction of gas chromatographic retention indices [19,20]. The position of features and single amino acids in a sequence severely affects CCS values [14,16]. This makes the application of a 1D CNN to this task more difficult. However, the problem can be solved using additional input features that provide a 1D CNN with information on global and relative positions of amino acids in a sequence.

The present study aims to develop a set of 1D spatial and tabular features that can be used with a 1D CNN for the prediction of peptide CCS values. These features will be used for investigating whether a 1D CNN can give comparable or better results in this task compared with an RNN, and for the evaluation of transferability of the developed model using an external test set acquired in different conditions compared with the training set. The resulting machine learning model and source code are published online.

## 2. Materials and Methods

### 2.1. Data Sets

The data set containing 718,917 CCS values was downloaded from the following link: https://github.com/theislab/DeepCollisionalCrossSection/blob/master/data/cobined_sm.csv.tar.gz (accessed on 16 November 2021). Reference [16] describes the data set. This combined data set contains two subsets: the main data set and the ProteomeTools data set. Following the authors of reference [16], we used the latter as a holdout test set. We shuffled the main data set and randomly split it into a training set (85%), validation set (5%), and test set (10%). Finally, we obtained the ProteomeTools test set (185,248 CCS values), test set (53,330 CCS values), validation set (74,666 CCS values), and training set (453,313 CCS values). The first two were neither used for the model development nor for training. There were no peptides simultaneously present in the training set and test sets. The validation set was used for the model development and accuracy monitoring during the training.

The data set was acquired using Bruker Daltonik timsTOF Pro instrument (Germany). The data set contains CCS data for the following charge states: + 2, + 3, and + 4. The data set contains tryptic peptides (most peptides in the data set have arginine or lysine at the C-terminus, but not all of them) and peptides obtained after digesting using Lyc-C, Lys-N proteases. Most peptides are not subjected to any modifications, and peptides with only two types of modifications are present in this data set: N-terminal acetylation and oxidation of methionine residue with the formation of a residue of methionine sulfoxide.

A data set from reference [13] was used for external testing. It was obtained using the IMS instrument of a different type and contains a significantly different set of tryptic peptides. Charge states other than + 2, + 3, and + 4 were removed (<2% of all data set), and the set of 8566 CCS values for unmodified peptides was finally obtained and later is referred to as the external test set. Peptides contained in both the training set and the external test set were removed from the training set. The data set sizes given above take this removal into account.

### 2.2. Input Features for the Deep Neural Network

Each amino acid residue by itself (besides features that characterize its position in a sequence or surrounding) was characterized by 45 features. The features are described in Table 1.

We denote these features as $F_{i}^{j}$, where *i* (0–44) denotes the feature type and *j* denotes the residue position in the peptide. Then, we considered the following sets of cumulative features that describe parts of the peptide:

(1) $$C_{i}^{1}\left(j_{start},j_{end}\right)=\frac{\sum_{j=j_{start}}^{j=j_{end}}F_{i}^{j}}{100}$$

(2) $$C_{i}^{2}\left(j_{start},j_{end}\right)=\frac{\sum_{j=j_{start}}^{j=j_{end}}F_{i}^{j}}{\sum_{j=0}^{j=n-1}F_{i}^{j}}$$

(3) $$C_{i}^{3}\left(j_{start},j_{end}\right)=\frac{\sum_{j=j_{start}}^{j=j_{end}}F_{i}^{j}}{j_{start}-j_{end}+1}$$

where *j*_start_, *j*_end_ are the positions of the first and last residues of the considered part of the peptide, and *n* is length of the peptide. Division by 100 was applied to avoid input values that are too large because deep learning frameworks cannot handle such values.

The peptide sequence was encoded as a fixed-length (more than 40 characters longer than the longest peptide in data sets) string that was padded with spaces. Each character encodes 1 residue, and an additional symbol was introduced for internal representation of oxidized methionine. We created a combined set of features for each character. The first one is 1 for each amino acid in a sequence and 0 for spaces. The next three features are binary features that identify whether this character denotes N-terminus (modified or not), acetylated N-terminus, or C-terminus. The next 315 features are $F_{i}^{j}$,$C_{i}^{1}\left(0,j\right)$, $C_{i}^{2}\left(0,j\right)$, $C_{i}^{3}\left(0,j\right)$, $C_{i}^{1}\left(j,n-1\right)$, $C_{i}^{2}\left(j,n-1\right)$, $C_{i}^{3}\left(j,n-1\right)$. These features characterize the amino acid and parts of the peptide that are located before and after it. The next three features explicitly characterize the position of the amino acid in the peptide: relative position, distance from N-terminus, and distance from C-terminus. The last feature characterizes the charge state of the peptide.

**Figure 1:**
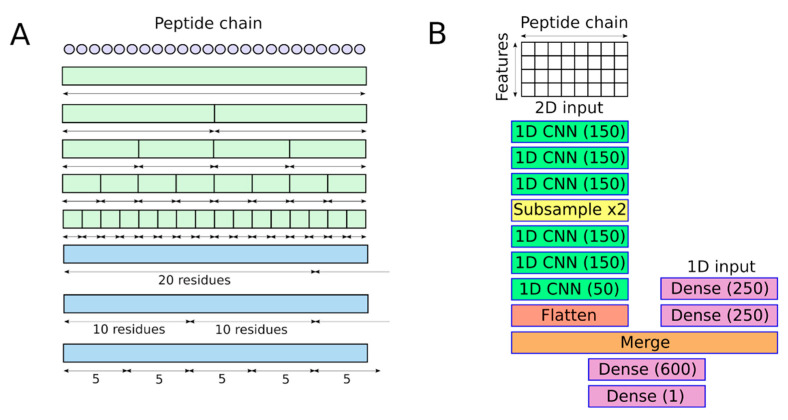
The deep neural network for CCS prediction and its input features: (**A**) subsequences used for the creation of input features for dense layers; borders of subsequences with non-integer length are rounded in such a way that at least one residue is included in each subsequence; (**B**) architecture of the neural network. Numbers in parenthesis denote the number of output channels or nodes (only for trainable layers).

In addition to the convolutional layers, a subnetwork consisting of dense (fully connected) layers was used. This subnetwork has separate input, and the following set of features was used. The first feature is the length of the peptide chain, the next four characterize charge state (integer and one-hot encoded), and the next one is a binary feature that specifies whether the N-terminus is acetylated. In addition to these six features, the feature set contains multiple $C_{i}^{1}\left(j_{start},j_{end}\right)$ features for various subsequences. Subsequences of fixed lengths (e.g., 5, 10, 20 amino acids) and subsequences with lengths equal to 1/2, 1/4, 1/8, and 1/16 of the peptide were considered. All subsequences considered for the creation of features for dense layers are depicted in Figure 1A.

### 2.3. Neural Network Architecture and Training

The neural network (NN) consists of two subnetworks. The first subnetwork consists of six convolutional layers. The last of them has 50 output channels (filters), and all other convolutional layers have an output of 150 channels. Between the third and fourth convolutional layers, a subsampling layer (kernel size and stride are 2) is connected. Kernel size and stride for all convolutional layers are 6 and 1, respectively. The last convolution layer is followed by a reshape (flatten) layer that rearranges the output matrix to a vector. The second subnetwork consists of two dense (fully connected) layers with 250 output nodes each. Outputs of both subnetworks are merged, and the merge layer is followed by two dense layers with 600 and 1 output nodes, respectively. All layers use the ReLU activation function except the output one. The last one uses a linear activation function. Figure 1B demonstrates the architecture of the NN.

All layers were trained together as a NN with two inputs and one output. The Adam optimizer was used with a learning rate of 0.0003 and batch size 256; the ReLU weight initialization algorithm was used. Mean absolute error (MAE) was used as a loss function. CCS values (Å^2^) were divided by 1000 before the training and, consequently, the prediction of the NN should be multiplied by 1000. The training was conducted using Java programming language and Deeplearning4j (1.0.0-M1) framework.

Pre-trained model parameters and source code are available online: doi.org/10.6084/m9.figshare.17080787 (accessed on 18 December 2021).

## 3. Results and Discussion

### 3.1. Accuracy of Collision Cross Section Prediction

**Figure 2:**
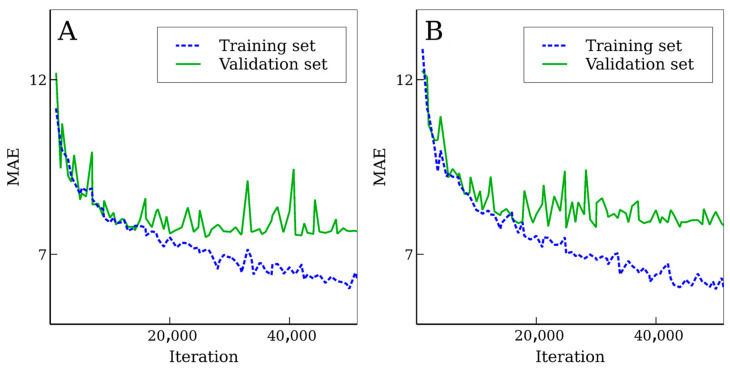
Learning curves (dependencies of accuracy on the number of training iterations) for (**A**) a neural network with the full feature set and for (**B**) a neural network with the reduced feature set.

A total of 50 training epochs (full data set runs) were performed. Every 1000 training iterations and after each epoch (1770 iterations), validation using the validation set was performed. The best validation set accuracy (in terms of MAE) was obtained after 26,000 iterations (~15 epochs). However, after 14,160 iterations (8 full epochs), accuracy was almost constant, and only a minor improvement in the validation set accuracy was achieved. The learning curve is demonstrated in Figure 2A. Accuracies of the NN after 26,000 and 14,160 training iterations were similar to each other for both test sets. Further training led to a minor improvement in terms of MdPE, but other error measures slightly increased. The best result in terms of MdPE for the validation set was after 43 epochs (76,110 training iterations).

Accuracies for the training set after 14,160 and 26,000 iterations were close to accuracies for the test and validation sets; hence, the NN did not experience significant overfitting. The NN after 76,110 (43 epochs) training iterations demonstrated much better accuracy for the training set compared with the test and validation sets due to overfitting. We used NN parameters obtained after 26,000 iterations for further evaluation. The difference in the accuracy between compounds that were and were not used for the training did not directly affect accuracy for unseen compounds and generalization ability; however, for the further development of scoring systems, a model that is not overfitted would be preferable.

The vast majority of input features are $C_{i}^{x}\left(j_{start},j_{end}\right)$ features (*x* = 1, 2, 3). Their generation takes negligible time compared with the NN processing. However, we trained the NN without these input features in order to study if these features can actually improve the accuracy. We refer to this NN as a NN using the reduced feature set. In this case, only six input features were left for dense layers, and we found that removing the first two dense layers improved the accuracy. Therefore, six global input features were merged directly with the output of the flatten layer (output of the convolutional subnetwork). The following accuracy measures were computed: root mean square error (RMSE), MAE, mean percentage error (MPE), MdPE, coefficient of determination (*R*^2^), Pearson’s coefficient (*r*), and the percentage error range that fits 90% of the values (Δ_90_). Accuracy measures for the training set, the test set, and the ProteomeTools test set are shown in Table 2. Accuracy measures for the validation set were very close those observed for the test set in all cases. Table 2 contains data for the NN with full and with reduced feature sets. Four models are presented there: the NN with the full feature set after 14,160, 26,000, and 76,110 training iterations and the NN with the reduced feature set. For the NN with the reduced feature set, the best accuracy for the validation set was observed after 17 epochs. The learning curve is shown in Figure 2B.

Table 2 shows that the use of $C_{i}^{x}\left(j_{start},j_{end}\right)$ improved the accuracy and the best results were achieved. Compared with the NN with the full feature set, the NN with the reduced feature set demonstrates worse accuracy for all considered test sets. We repeated the training five times, and this was observed every time. Uncertainty linked with inaccurate reproducibility of training (initial weight initialization and the optimization algorithm are stochastic) was 0.01 percentage points in terms of MdPE.

### 3.2. Comparison with Previous Works

**Figure 3:**
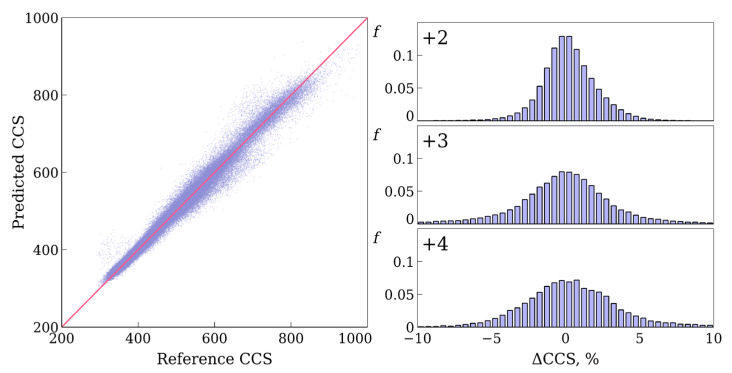
A correlation plot between reference and predicted using a deep neural network CCS values (Å2) and distribution of relative errors (%) between reference and predicted CCS values for various charge states.

The results of the accuracy evaluation and comparison with the previous work [16] are given in Table 3. Our model outperformed the previously published RNN for the ProteomeTools test set. An equal or better performance was observed for all charge states (separately), for all ranges of CCS values and all considered accuracy measures. It can be concluded that the 1D CNN gives an appropriate accuracy for the prediction of CCS values for peptides and can be used instead of the RNN or together with the RNN as part of an ensemble of models. An ensemble of independent models is usually more accurate than each of the models [19,20,23]. The averaging of the results of various models improves the accuracy [20]. Figure 3 demonstrates a correlation between the predicted and reference values and error distributions for different charge states.

Both NN architecture and the features set affected the accuracy of the model. In work [16] a raw peptide sequence and charge state were used as input features without any feature engineering. Such an input is close to our reduced feature set but is even more minimalistic. We trained the CNN with such input features (architecture was the same as that of NN with a reduced feature set). The MAE, MdPE, Δ90, and *r* values were 9.9 Å^2^, 1.36%, 4.24%, and 0.991, respectively, for the ProteomeTools test set. Additionally, we trained the RNN with architecture similar to that of the RNN from work [16], but using a full feature set developed in this work. In this case, convolutional, subsampling, and flatten layers were replaced with two bidirectional LSTM (long short-term memory) layers. The number of layers and nodes was the same as in work [16], and the last time frame layer was used after LSTM layers. MAE, MdPE, Δ_90_, *r* values were 9.6 Å^2^, 1.31%, 4.08%, and 0.991, respectively for the ProteomeTools test set. Therefore, the best results were achieved only when the full feature set was used with CNN. Only simultaneous use of the full feature set and CNN architecture improved the accuracy in terms of the Δ_90_ value.

In order to evaluate if this model is applicable for other data, an external test set was used. The training set consisted of peptides obtained by the digestion of proteins extracted from the human HeLa cell line and four other organisms (Caenorhabditis elegans, Drosophila melanogaster, Escherichia coli, and Saccharomyces cerevisiae). The external test set consisted of peptides obtained by the digestion of proteins from human blood plasma, mouse blood plasma, and Shewanella oneidensis. The external test set contains 8566 peptides, and only a few hundred of them were in the training set. We excluded these peptides from the training set before all training operations described in this work. However, a relatively low fraction of peptides is contained in both data sets simultaneously. The training set was acquired using a trapped ion mobility spectrometer, and the external test set was acquired using a drift tube ion mobility spectrometer. Therefore, it can be concluded that these two data sets are quite different in nature, and the external test set can be used to evaluate the generalization ability of the model.

**Figure 4:**
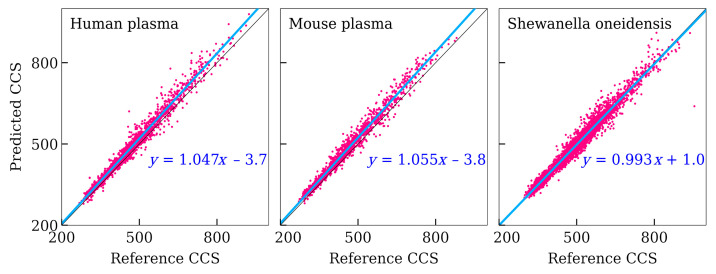
Correlation plots between reference and predicted CCS values (Å^2^) for peptides from various organisms (external test set).

The MAE, MdPE, Δ_90_, and *r* values were 16.0 Å^2^, 2.73%, 7.10%, and 0.984, respectively. This accuracy is still satisfactory but worse compared with previously considered test sets. Table 4 shows the accuracy of CCS prediction for peptides extracted from various organisms separately. The correlation coefficient and the coefficient of determination are almost the same for these three organisms. However, other accuracy measures are much worse for human plasma and mouse plasma. This could be caused by a systematic error. Figure 4 shows correlations between the predicted and reference values, and there is a significant systematic error for human plasma and mouse plasma. Most likely, this error was introduced during the computation of CCS values by the authors of reference [13].

In reference [13], the accuracy of their drift time prediction model was given for the data set used in this work as the external test set. The same data set was used for the training and testing by the authors of that work. Accuracy values are given in terms of *R*^2^ and mean square error (MSE) for drift times. Our model predicts CCS values, not drift times, and we should compute drift times using CCS values. The following equation was used:

(4) $$t_{d}=A\Omega \mu /z+B$$

where *t_d_*—drift time (s), Ω—the value of CCS (Å^2^), *A* = 0.0248 and *B* = 2.15 are parameters that depend on parameters of IMS system (e.g., pressure, length, voltage, etc.), *z*—charge state, *μ*—the reduced mass of ion (*μ* = *mM*/(*M* + *m*), where *m*—mass (Da) of the neutral gas molecule and *M*—mass (Da) of the ion). If only R^2^ values are used as an accuracy measure, the results do not depend on *A* and *B* values. These values can be calculated from parameters of the system or fitted using reference drift times and predicted CCS values. We used the second way due to a systematic error in reference data. The comparison with the accuracy of the drift time prediction model from work [13] is shown in Table 5. Our model provided better predictions for all charge states but + 4. Therefore, it can be concluded that our NN has a sufficiently good transferability and generalization ability to predict CCS and drift times for a completely different set of peptides with satisfactory accuracy.

Work [13] was selected for external testing for several reasons. The authors provided a data set containing CCS values; only the CCS values (not drift times or other values) are transferable and system independent. Work [13] provides accuracy measures in a form that can be used for direct comparison. It considers large and diverse enough data sets with sufficiently long peptides measured in charge states + 2, + 3, + 4. For small data sets with very specific data, models that are specialized for such data can outperform models that are more versatile; hence, only comparison using large enough data sets is meaningful.

### 3.3. Model Averaging for Further Improvement of Accuracy

Further improvement in predicting accuracy is possible using a few independently trained NN of the same architecture. The trainable parameters of NN (weights and biases) are initialized randomly at the beginning of the training, and the Adam optimization algorithm is stochastic. Hence, the final set of parameters is different every time the model is trained, and the errors of the model for each peptide are random too. Using this fact, a simple way to improve the predicting accuracy is training several NN of the same architecture and averaging predictions.

The training of each NN takes ~11–12 h (30,000 training iterations). Prediction of CCS for 185,248 peptides using a single NN takes 343 s (1.9 ms per peptide). These data are given for a single computer equipped with a single Nvidia 2070 GPU and an Intel i5-8400 6-core central processor (CPU). If GPU is not used, and prediction is made only on CPU, a prediction rate is ~3 ms per peptide using a single NN. Therefore, using an ensemble of NN of the same architecture is a computationally suitable way to improve accuracy.

**Figure 5:**
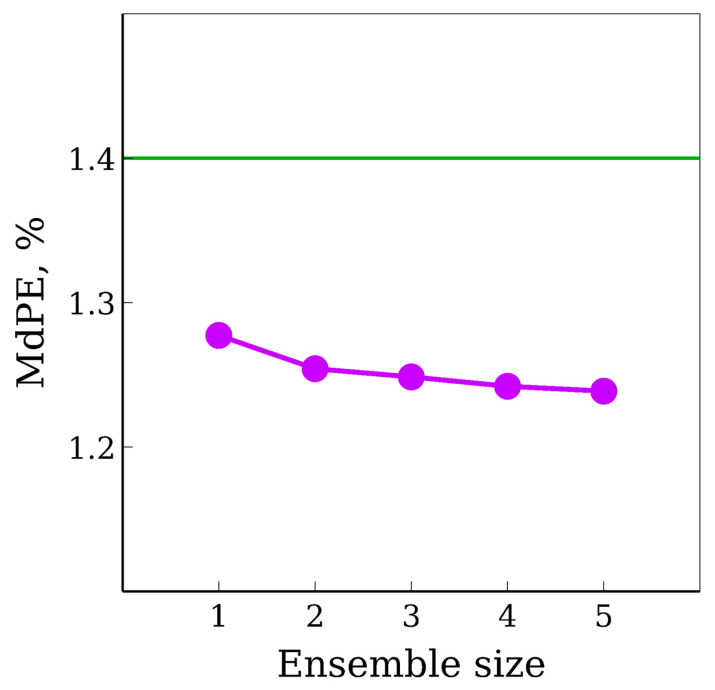
The dependence of median percentage error on the number of neural networks of the same architecture in the ensemble. A horizontal line denotes the accuracy of the previous model described in reference [16].

The ensemble of 5 NN of the same architecture resulted in the following accuracy for the ProteomeTools test set: RMSE, MAE, MPE, MdPE, Δ_90_, R^2^, and *r* values were 14.2 Å^2^, 9.0Å^2^, 1.77%, 1.24%, 3.87%, 0.984, and 0.992, respectively. The dependence of MdPE on the number of NN in the ensemble is shown in Figure 5.

The most significant accuracy measures for the further development of scoring systems are MdPE and Δ_90_, while *r* and *R*^2^ are convenient but not relevant for such application. Relatively small amounts of large errors have the biggest impact on such common accuracy measures as *r* and *R*^2^, and RMSE. Some improvement in terms of the most relevant accuracy measures was achieved using an ensemble of CNN and the developed feature set compared with previous work.

## 4. Conclusions

A 1D convolutional neural network is an accurate method of prediction of collision cross sections of peptides based on their sequence. The developed neural network is more accurate compared with the previously described recurrent neural network. The achieved median percentage error was 1.28%, and the root mean square error was 14.6 Å^2^. The developed model was tested using the external test set, and reasonable accuracy was observed. This proves the generalization ability of the neural network. Averaging predictions of several neural networks of the same architecture, which were trained independently, allows the accuracy to be further enhanced. The developed models can be used in ensembles with models of different nature (such as recurrent neural network) in future research. It was previously shown [19] that the simultaneous use of various models using different inputs greatly decreases error. The feature set developed in this work can also be used in further research. The pre-trained parameters of models and source code are available in the online repository: doi.org/10.6084/m9.figshare.17080787 (accessed on 18 December 2021).

## Figures and Tables

**Table 1:** Features (descriptors) that characterize a single amino acid residue. Letters in parentheses denote amino acid residues for which the considered feature is 1.

*N*	Description of Features
0–20	One hot encoded amino acid (including oxidized methionine as the 21st amino acid). One of these features is set as 1 and others are set as 0.
21–25	Elemental composition of the residue: integer values that describe a number of each element (H, C, N, O, S).
26–31	Six binary features that show whether the side-chain of the amino acid is acidic (D, E), modified (non-standard), has an amide group (N, Q), is non-polar (G, A, V, L, I, P, M, F, V), is small (P, G, A, S), is uncharged polar (S, T, N, Q, C, Y, oxidized methionine residue).
32–35	Four binary features that show whether the side-chain of the amino acid is aliphatic non-polar (V, I, L, G, A), is aromatic (W, F, Y), is positively charged (K, R, H), has a hydroxyl group (S, T, Y).
36	For N, D (Asp, Asn) this feature is 1, 0 elsewhere.
37	For E, Q (Glu, Gln) this feature is 1, 0 elsewhere.
38–43	Descriptors of amino acids from references [21,22], six float values, constant for each amino acid type.
44	Always 0 for all amino acids, 1 for padding symbol.

**Table 2:** Accuracy measures after various numbers of training iterations for two different feature sets and three data sets.

Data Set	Training Iterations	Features	Accuracy Measures						
			RMSE, Å^2^	MAE, Å^2^	MPE,%	MdPE, %	Δ_90_, %	*R* ^2^	*r*
Training set	14,160	Full	12.6	7.5	1.46	1.00	3.21	0.987	0.993
	26,000	Full	11.7	7.1	1.40	0.96	3.05	0.988	0.994
	76,110	Full	9.8	6.2	1.23	0.87	2.68	0.992	0.996
	30,000	Reduced	11.4	7.0	1.38	0.95	3.03	0.989	0.994
Test set	14,160	Full	13.1	7.7	1.50	1.02	3.28	0.985	0.993
	26,000	Full	12.8	7.6	1.47	1.00	3.25	0.986	0.993
	76,110	Full	13.2	7.6	1.48	0.99	3.22	0.985	0.993
	30,000	Reduced	13.3	7.8	1.52	1.03	3.32	0.985	0.992
ProteomeTools test set	14,160	Full	14.9	9.4	1.84	1.28	4.02	0.983	0.991
	26,000	Full	14.6	9.3	1.82	1.28	3.95	0.983	0.992
	76,110	Full	15.3	9.5	1.86	1.27	4.06	0.982	0.991
	30,000	Reduced	15.4	9.7	1.90	1.32	4.17	0.982	0.991

**Table 3:** Accuracy for different charge states and CCS ranges for the neural network with the full set of features after 26,000 training iterations and the ProteomeTools test set. The values from reference [16] are given with the same accuracy as in the corresponding work.

Subset	This Work			Previous Results [16]		
	MdPE, %	Δ_90_, %	*r*	MdPE, %	Δ_90_, %	*r*
Full ProteomeTools test set	1.28	3.95	0.992	1.40	4.0	0.992
Charge state + 2	1.06	3.06	0.985	1.2		
Charge state + 3	1.77	5.49	0.938	1.8		
Charge state + 4	1.95	5.47	0.886	2.0		
CCS values 0–400	1.06	3.02	0.920	1.2		
CCS values 400–800	1.38	4.32	0.986	1.5		
CCS values 800–1200	2.30	5.71	0.695	2.2		

**Table 4:** Accuracy for different subsets of the external test set.

Subset	This Work					
	RMSE, Å^2^	MAE, Å^2^	MdPE, %	Δ_90_, %	*r*	*R* ^2^
Human plasma	26.7	20.4	3.85	7.99	0.989	0.979
Mouse plasma	26.9	21.7	4.53	8.55	0.990	0.980
Shewanella oneidensis	17.2	11.8	1.85	5.18	0.988	0.976

**Table 5:** Accuracy of drift time prediction for different charge states for the external test set in comparison with the results from reference [13].

Subset	This Work		Previous Results [13]	
	*R* ^2^	MSE, s^2^	*R* ^2^	MSE, s^2^
Charge state + 2	0.9634	0.287	0.9620	0.290
Charge state + 3	0.9179	0.715	0.9062	0.813
Charge state + 4	0.9266	0.705	0.9308	0.556

## Data Availability

The data used for model training and testing are available via corresponding references. Source code and pre-trained model parameters are provided in the online repository: doi.org/10.6084/m9.figshare.17080787.
